# A Protocol for Evaluating the Effectiveness of an Integrated Group Postpartum and Well-Child Care Model on Maternal and Child Health Outcomes

**DOI:** 10.3390/healthcare14213751

**Published:** 2026-11-03

**Authors:** Ashley Gresh, Esnath Kapito, Janet Mambulasa, Nellie Ngutwa, Nancy Perrin, Mina Kazemi, Rheanna Platt, Crystal L. Patil, Ellen Chirwa

**Affiliations:** 1 School of Nursing, Johns Hopkins University, 525 North Wolfe Street, Baltimore, MD 21205, USA; nperrin@jhu.edu (N.P.); mkazemi2@jhmi.edu (M.K.); 2 School of Maternal, Neonatal and Reproductive Health, Kamuzu University of Health Sciences, Private Bag 360, Chichiri, Blantyre 3, Blantyre P.O. Box 415, Malawi; ekapito@kuhes.ac.mw (E.K.); jmambulasa@kuhes.ac.mw (J.M.); nngutwa@kuhes.ac.mw (N.N.); embweza@kuhes.ac.mw (E.C.); 3 School of Medicine, Johns Hopkins University, 733 N. Broadway, Baltimore, MD 21205, USA; rplatt1@jhmi.edu; 4 Department of Health Behavior and Clinical Sciences, School of Nursing, University of Michigan, 426 N Ingalls St, Ann Arbor, MI 48109, USA; clpatil@umich.edu

**Keywords:** postpartum health, infant health, health care service delivery

## Abstract

Background/objectives: The building and then testing of high-quality maternal and infant health care service delivery models are urgently needed to address the persistent maternal and infant health disparities globally. A range of health system and social drivers contribute to maternal and infant morbidities and mortality, including limited access to care and preventive services and variation in care quality. These poor outcomes have led to calls for a broader redesign of maternal and infant health services and delivery systems. One promising model is group care, which has shown potential for maternal and child health care redesign, but has been studied mainly during the antenatal period. This paper describes the study protocol our team will use to evaluate the effectiveness of group postpartum and well-child care. Methods: We will conduct a cluster randomized controlled trial with mixed methods across 16 clinics in Zomba District, Malawi to test the model’s impact on select maternal and infant health outcomes. We hypothesize that compared to usual care, women in the intervention group will have decreased postpartum depression symptoms, anemia, and hypertension and infants will have increased vaccination rates and more optimal secondary outcomes for the dyad, such as uptake of family planning, exclusive breastfeeding, childhood development (gross and fine motor skills, social-adaptive development, and nutrition). We will also qualitatively explore clinic- and patient-level implementation facilitators and barriers for group care and the health and social needs that arise in the 12 months after birth for both women and their infants in both arms. Discussion: This study will be the first to conduct an adequately powered trial of a group postpartum and well-child care model in a low-resource setting. Findings from the study will contribute important evidence on how postpartum and well-child care may be improved during the first year after birth. Trial registration: ClinicalTrials.gov registration number: NCT07414901.

## 1. Introduction

There are alarmingly high rates of maternal and infant morbidity and mortality, particularly in low-income countries. These outcomes are largely preventable through earlier identification and treatment of health issues along with high-quality health care across the care continuum. Malawi, a low-resource country in sub-Saharan Africa, continues to experience some of the worst maternal health outcomes globally. Each year, > 224 women per 100,000 live births and 35 infants per 1000 live births die [1]. While national maternal morbidity rates are unavailable for Malawi, rates in Sub-Saharan Africa are estimated to be 198 per 1000 live births [2]. Pregnancy-related complications and maternal morbidities such as perinatal mental health disorders, uncontrolled hypertension, and infection can reduce quality of life and increase mortality risk during the extended (e.g., up to 12 months after birth) postpartum period [3,4,5]. If left untreated, these unaddressed maternal morbidities not only impact the postpartum person but can also directly impact infant health and development, for whom the first year of life is critical, and can set the stage for long-term health outcomes [6,7]. In Malawi, a range of structural health system and social factors contribute to maternal and infant morbidities, including low rates of receipt of health care and preventive services and variable quality of care [8]. To address this, there have been calls for a redesign of perinatal and infant health services [9].

The World Health Organization (WHO) recommends that every postpartum person and infant have at least four visits within the first six weeks postpartum regardless of where they gave birth [10]. While the Malawian Ministry of Health adheres to these guidelines and recommendations, approximately 50% of women who gave birth at a medical institution did not receive any postpartum care at the recommended 48 h, 1-week, and 6-week postpartum visits [11]. These missed visits indicate the need for strategies to improve access to postpartum care and extend that care beyond six weeks after birth to fill the gap in the maternal and child health care continuum in the first year after birth. Not reaching women in this critical period leads to missed opportunities for identifying and managing health and social challenges and promoting healthy behaviors for both the woman and the infant [12].

Group care is a promising approach for redesigning maternal and child health services but has primarily been tested in antenatal care. Studies show the feasibility and effectiveness of bringing group antenatal care to scale in low-resource settings and demonstrated increased antenatal care visit attendance, facility-based births, and health literacy outcomes [13,14]. Despite the potential benefits, few studies have examined implementing group care in the postpartum period, integrating postpartum and pediatric care in low- and middle-income countries (LMICs) [15]. The year after birth offers an important opportunity to address ongoing maternal and infant health and social needs while supporting the transition of the dyad from postnatal to primary care. Preliminary findings suggest that Centering-based group well-child care (CenteringParenting is the predominant model of group prenatal and group well-child care) is a family-centered and efficient model that improves clinical outcomes [16]. Group care beyond birth brings together 8–10 postpartum people with similarly aged infants to receive care together [17]. Each visit is approximately 2 h, with the first 30–45 min focused on clinical health assessments for both the postpartum person and the infant and participation in self-assessments such as taking one’s own blood pressure and weight. The remaining 75–90 min are devoted to facilitated, interactive health promotion and education activities. Although the group well-child care model has shown promise [16], it has not been widely implemented nor evaluated in LMICs such as Malawi. Group well-child care is often focused solely on the infant without integrating postpartum care, and no studies to our knowledge have conducted a clinical trial and evaluated its impact on both maternal and child health outcomes.

To address the gap in research our research team co-designed and piloted an integrated group postpartum and well-child care (GPWCC) model in Malawi. The pilot showed this model was highly acceptable, appropriate, and feasible for women and health care workers and that clinics could implement group sessions with fidelity [18,19]. This six-visit model across the infants’ first year aligns with the infant vaccination schedule and includes Malawi-specific health promotion content for the dyad. However, pilot studies cannot establish effectiveness. This study will use a cluster randomized controlled trial to evaluate the effectiveness of the group care model across clinics in Zomba District, Malawi. This paper describes the study protocol our team will use for the effectiveness evaluation of GPWCC. This protocol has been approved by the Johns Hopkins Medicine and Kamuzu University of Health Sciences Institutional Review Boards.

## 2. Methods

### 2.1. Study Aims

The specific aims are:

**Aim 1.** Evaluate the effectiveness of GPWCC through 12 months postpartum. We hypothesize that compared to usual care, women in group care and their infants will have:

**H1.**  *Increased infant vaccination rates (co-primary outcome).*

**H2.**  *Decreased postpartum depression (co-primary outcome), anemia, and hypertension.*

**H3.**  *More* *optimal secondary outcomes for the postpartum person and infant with regard to uptake of family planning, exclusive breastfeeding, childhood development, and nutrition.*

**Aim 2.** Explore the health and health-related social needs that arise in the 12 months after birth for both women and their infants.

**Aim 3.** Identify clinic- and patient-level group care implementation facilitators and barriers.

**Figure 1:**
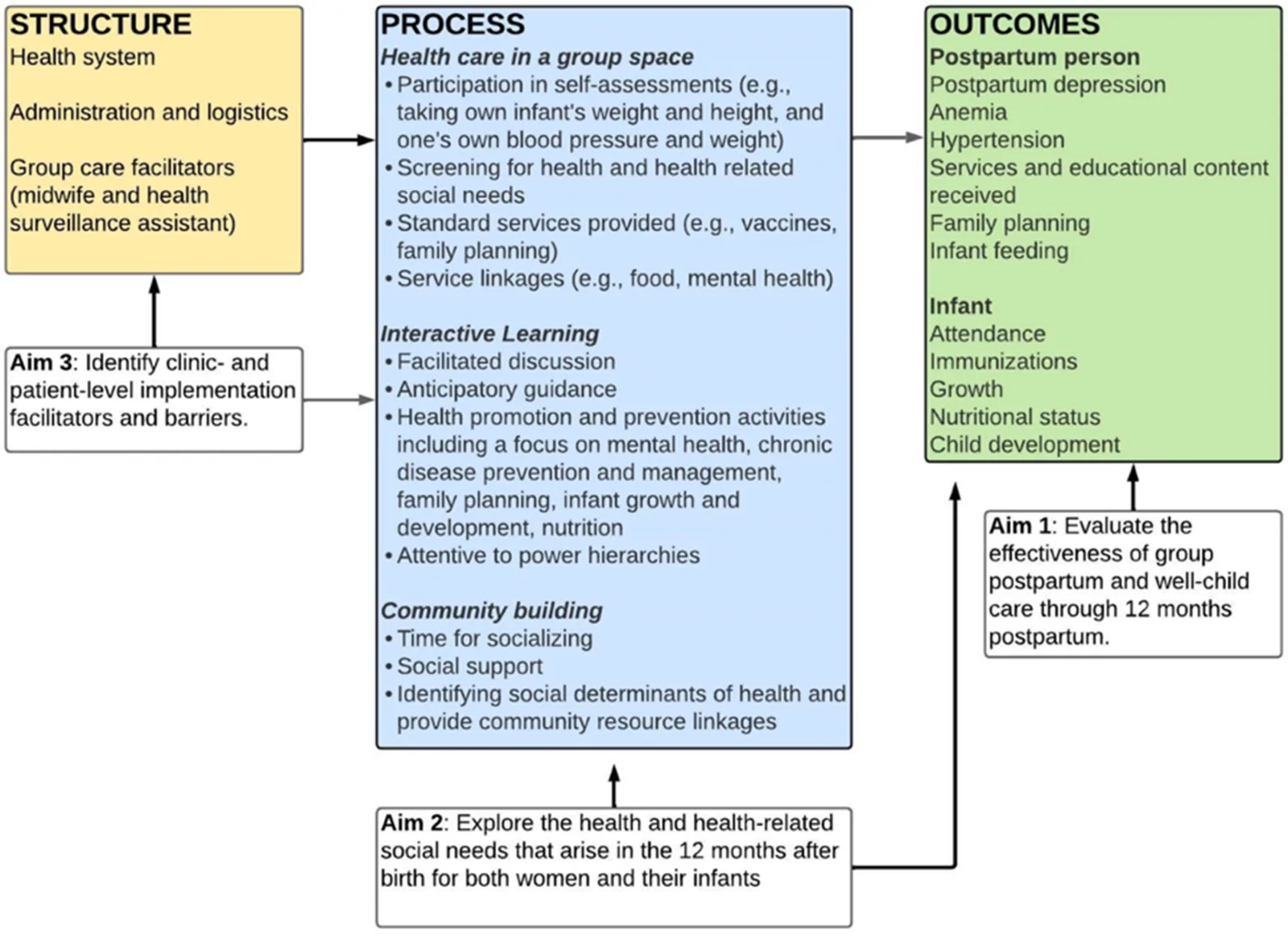
Conceptual framework for group postpartum and well-child care based on Donabedian’s quality of care framework [20].

The Donabedian quality of care framework [20] guides this study. The framework hypothesizes that health care structure and process lead to improved outcomes. Figure 1 shows how the group care model’s structure and process (the three core components) are hypothesized to improve outcomes for the dyad.

### 2.2. Study Design Overview

**Figure 2:**
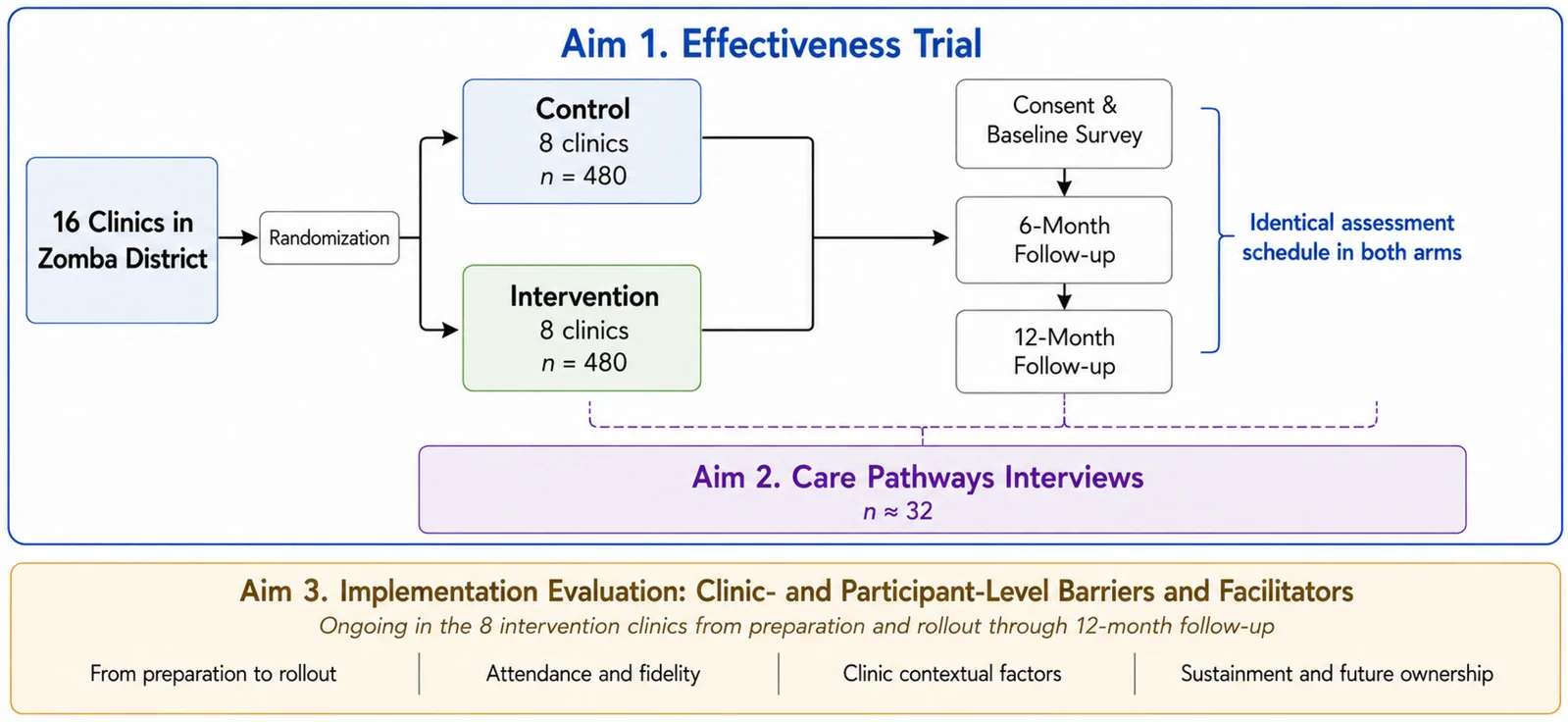
Project aims and overview.

A cluster randomized controlled trial design with mixed methods will be used to determine the impact and comparative effectiveness of group versus usual individual care for postpartum women and their infants in 16 clinics across Zomba District, Malawi (see Figure 2 for the overview of the study design). Data will be collected at baseline, 6 months and 12 months. Women will be nested within the postpartum/well-child care group for the intervention. In the control arm, dyads will be grouped based on order of entry within each site. The first 10 dyads will form “group 1,” the second 10 dyads “group 2,” etc. Integration of quantitative and qualitative findings will occur at the interpretation stage, where qualitative data on health and social needs (Aim 2) and implementation barriers and facilitators (Aim 3) will be used to contextualize the effectiveness findings (Aim 1) using an explanatory sequential mixed methods approach.

### 2.3. Study Setting

The study will be conducted in Zomba District in Southern Malawi, where rates of maternal and infant morbidities and mortality remain high. Partnering clinics in neighboring Blantyre District have established a group care training program with eight trained facilitators and technical advisors to support group care implementation in Zomba District.

### 2.4. Clinic Assessment and Selection

Sixteen health facilities in Zomba Health District were selected based on the (1) number of postnatal visits monthly, (2) infant vaccination rates monthly, (3) staffing, and (4) clinic willingness to participate in the study. These clinics represent a wide variation in size and staffing in Malawi and include urban, peri-urban, and rural locations. This sample of clinics allows us to assess whether government-run postnatal and well-child care clinics can implement group care under everyday clinic conditions without major resource shifts. This will provide critical information for the scale-up and sustainability of the model.

### 2.5. Randomization

To help balance the two arms, the 16 clinics were ranked based on clinic size. Clinics were then 1:1 randomized to either integrated GPWCC (intervention) or routine postpartum and well-child care (control) in block sizes of two. The locations of the chosen clinics ensure that participating clinics will be far enough apart to minimize the likelihood of cross-group contamination. In Malawi, women typically attend their nearest health facility for postnatal and well-child care, making crossover between study clinics unlikely. Health care workers at participating clinics are assigned to their facility and do not rotate between study sites.

Control Group: Individual care (usual): Congruent with WHO recommendations and Malawian national health guidelines, individual postnatal care consists of four visits: at 24 h, 48–72 h, 1-week, and 6 weeks after birth. Women are provided postnatal care services on a first-come, first-served basis and listen to a health lecture. They meet individually with a midwife for a physical assessment. There are no guidelines for infant well-child care in Malawi; however, health care workers do use the WHO Integrated Management of Childhood Illnesses and Emergency Triage, Assessment and Treatment guidelines and follow a recommended childhood vaccination schedule. When infants come to get vaccinated, they are also weighed and measured for growth monitoring by health surveillance assistants (HSAs). The vaccination schedule in the first 12 months is recommended at 6, 10, and 14 weeks of age and at 6, 9, and 12 months of age.

Intervention Group: Group care: Women will have additional visits compared to those in usual care. Their first postnatal care visits are conducted individually during the usual recommended 24 h, 48–72 h and 1-week postnatal visits. This will be identical to usual care. It is not feasible to have group visits before 6 weeks postpartum because it corresponds with a woman’s delivery date, and in the co-creation process in our previous work, women did not want to be in a group visit this early after birth [19]. After the 1-week postnatal visit, women in the intervention clinics will then be given their group visit appointment schedule for all six sessions (see Table 1 for sessions, timeline, and topics for interactive learning). While the length of the visit can be a barrier to participation, group care is designed to integrate multiple visits into one visit to reduce the number of visits in the first year after birth. For example, our pilot demonstrated that one visit can include postpartum, well-child, vaccination, and family planning services. We purposively stagger the initiation of group care. This ensures that the implementation team is available to provide intensive interactive assistance. The GPWCC intervention includes multiple embedded components: (1) a team approach to integrated care (midwife and HSA); (2) increased contact frequency (increased number of postnatal visits); (3) resources provided for screening (scales, thermometers, tape measures); (4) integration of postpartum depression screening into routine care; (5) appointment reminders for group sessions; and (6) extended time for health education and peer support opportunities.

### 2.6. Data Collection and Analysis

#### 2.6.1. Aim 1: Evaluate the Effectiveness of GPWCC Through 12 Months Postpartum

Recruitment: Women and infant dyads in both intervention and control clinics have the same study intake visit at their 1-week postnatal visit. The research team will obtain consent to assess eligibility of interested women/infant dyads. All women presenting for their 1-week postnatal visit are approached sequentially and offered participation; the research team will not selectively recruit based on perceived interest. The number of women screened, eligible, and enrolled will be recorded at each clinic and reported by study arm. To be eligible, women must be 15 years or older and have had a singleton birth with an infant less than 4 weeks after birth at the time of recruitment. Eligible women in the intervention and control clinics will be provided detailed information about the study for informed consent. For participants aged 15–17 years, written informed consent is obtained from a parent, guardian, or legal representative, and written assent is obtained from the adolescent participant. Both the parent/guardian and adolescent sign the same consent/assent form. Participants aged 18 years and older provide independent informed consent. All consent and assent procedures were approved by both the Johns Hopkins Medicine Institutional Review Board and the Kamuzu University of Health Sciences Research Ethics Committee in accordance with local legal and regulatory requirements and ethical guidelines. Consenting women/infant dyads in the intervention clinics will be enrolled in GPWCC groups of 8–10 dyads based on the order of enrollment, with a total of six GPWCC groups per clinic staggered over time in the eight intervention clinics for a total of 480 dyads. We will continue recruiting until we have enough for the groups. However, we expect a 20% attrition rate, for an estimated sample of 384 dyads in the intervention clinics. The process will be used in the control clinics, with the first 8–10 dyads in the first group at each control clinic, with a total of six groups in each of eight control clinics, for a total of 480 dyads in the control clinics, again with an estimated 20% attrition for an estimated sample of 384 dyads in the control clinics. The intervention and control groups will therefore be similar on infant age and date of enrollment. Therefore, the anticipated final sample is 768 dyads across the 16 intervention and control clinics after taking into account attrition.

Retention: To maximize retention in the effectiveness evaluation across the three data collection time points (baseline, 6 months, 12 months), we obtain each woman’s cell phone where she can be reached, her house location, and two additional contacts, with cell phones where possible. Each effectiveness team member has a caseload of specific women and follows those women throughout the study. This continuity builds relationships and should enhance retention. The team member makes calls to set up and confirm data collection appointments and follow up if the woman misses her appointment. If a woman misses a data collection appointment and cannot be reached, the HSA assigned to that clinic will go into the community and attempt to locate the woman. This approach integrates lessons learned from our pilot and group prenatal care randomized controlled trial, including limited cell phone access among many of the rural women in Malawi [21]. We anticipate that these additional strategies, especially assistance from the HSA, will substantially increase retention. All these strategies are provided for data collection from all women in both study conditions. The research team will also provide attendance reminders for those in the group care intervention arm.

Data collection procedures: Each woman will complete a baseline survey administered by the study team orally and enter each woman’s responses on a tablet. These are completed off-line and transferred securely over the internet using REDCap.

Measures: Table 2 summarizes the outcomes selected to evaluate the impact of GPWCC. Outcomes are measured across three time points, which include data from self-reported surveys, the woman and infant’s health record, and two biomarker tests. Survey measures were selected from those used in research for group antenatal care, and most have been used with similar populations and have shown high reliability [22,23]. The study team will extract data from the health passport and take a photo of it to ensure accurate data capture. Photos will be deleted after data are inputted.

Many of the instruments have already been translated and validated in Chichewa (denoted in Table 2). For example, the Edinburgh Postnatal Depression Scale (EPDS) to screen for postpartum depression and the Malawian Developmental Assessment Tool (MDAT) to measure infant development have both been validated in the Malawian context [22,24]. All other instruments have been translated into Chichewa using a team translation approach with a translation and back translation of each survey by a translator.

Operational definitions for clinical outcomes are as follows. Infant immunization status is obtained from health records; “all recommended immunizations” refers to the Malawi vaccination schedule at 6, 10, and 14 weeks of age and at 6, 9, and 12 months of age. Hypertension is defined as blood pressure ≥140/90 mmHg or current use of antihypertensive medication. Anemia is defined as hemoglobin <11 g/dL for mothers and <10.5 g/dL for infants, consistent with WHO thresholds for postpartum women and children 6–59 months. Postpartum depression is defined as “a type of depression that causes intense feelings of sadness, anxiety, or despair that keep people from being able to do their daily tasks” [25].

Women’s hemoglobin (Hb) levels are measured at baseline and 12 months. Infant Hb will be assessed at 12 months. Hemoglobin levels are measured using a HemoCue^®^ 801 Hb point-of-care test. Hb values and health records will be recorded by the research team in the participant’s health passport (as appropriate) and on data collection forms developed for the research project, using the participant’s project ID and not her name. We have detailed the safety and health protocols, including referrals that provide resources to participants beyond the capacity of the study team. Participants with clinical findings requiring follow-up, including any positive response to the EPDS self-harm item (item 10), blood pressure ≥160/110 mmHg, or hemoglobin <7 g/dL are immediately referred to on-site midwives for clinical evaluation and management or further referral as needed. These safety protocols are applied uniformly in both study arms.

**Table 2:** Summary of maternal and child health outcomes being measured.

Construct	Operational Measure	Source *	Time Points
Maternal Outcomes			
Postpartum depression (primary outcome)	Edinburgh Postnatal Depression Scale ** [26] 10 Items	S	All
Family planning	Using a family planning method, yes/no	H, S	6 and 12
Content of care	Recall of services and educational topics covered in the postpartum	H, S	All
Behaviors			
Breastfeeding self-efficacy	Breastfeeding Self-Efficacy Scale ** [27]	S	All
Exclusive breastfeeding	Exclusive breastfeeding at 6 months	S	6
Maternal nutrition	Dietary Diversity for Women 8-item survey [28]	S	Baseline and 12 months
Biological			
Anemia	Hemoglobin (Hb), Point-of-care bloodspot, HemoCue^®^ (Hb < 11//dL) [29]	T	Baseline and 12
Hypertension (HTN)	3 categories: (1) Hypertension, on treatment; (2) No hypertension, no treatment; (3) Hypertension, no treatment	H, S, T	All
Infant			
Immunization (primary outcome)	Received all recommended immunizations yes/no	H	6 and 12 months
Growth	Weight for age (growth monitoring)	H	All
Nutritional Status	Mid–upper arm circumference (MUAC)	T	6 and 12
Anemia	Hemoglobin (Hb), Point-of-care bloodspot	T	12
Child Development	Malawian Developmental Assessment Tool ** (MDAT)	O	6 and 12

* Source: H = health record; S = self-report survey; T = medical test or measure, O = observation. ** Validated in Chichewa.

Analyses for Aim 1. Before any analyses are carried out, the data will be audited for quality and completeness and the distributions will be validated with reference to planned analysis models. The evaluation of distributions will include the detection of outliers in quantitative data and checking distributions of variables to ensure that they meet the assumptions of the planned analytical method. Reliability will be estimated (item analysis, Cronbach’s a) for scales in this sample. T-tests and chi-square analysis will be used to evaluate group equivalence at baseline. Baseline characteristics will be summarized descriptively by study arm. Prespecified covariates including maternal age, parity, baseline EPDS score, and infant age at enrollment will be included in all models. All analyses will follow intent-to-treat principles. Management of Missing Data: The pattern of missingness will be examined and baseline responses compared between those with and without missing data. Variables related to missingness will be included in the analyses, which should yield valid inferences. Multiple imputation will be used to handle missing data in the main analyses. We will complete sensitivity analyses comparing parameter values from the analyses with completers only with parameter values for analyses using multiple imputation to assess any bias introduced through the use of imputation. Should a clinic withdraw from the study, data from participants already enrolled at that clinic will be retained in analyses consistent with ITT principles. Mixed-effects models will be used to account for the hierarchical and longitudinal structure of the data with random effects for time (for outcomes measured repeatedly), postnatal care group, and clinic. To evaluate the effectiveness of GPWCC, logistic mixed-effects models will be used to assess whether or not the infant received all recommended vaccines at 12 months (primary outcome) and mother’s anemia at 12 months. Infants/mothers will be nested within groups and groups nested within clinics. Randomization arm will be a fixed effect at the clinic level. Multinomial mixed-effects regression will be used to assess mother’s hypertension at 12 months, measured as none; hypertension, on treatment; and hypertension, no treatment. Mixed-effect logistic models will be used to test differences over time (baseline, 6 and 12 months) in mothers screening positive for depression symptoms (EPDS ≥10) (primary outcome), as this threshold reflects clinical screening practice and aligns with the intervention’s goal of identifying and supporting women at risk. A secondary analysis using continuous EPDS scores will also be conducted using linear mixed-effects models to examine intervention effects across the full range of depressive symptoms. Randomization arm, time and the arm by time interaction will be included in the model. A significant arm by time interaction would signify that change over time in depression differed for the intervention and control arms. Significant interactions will be graphed for interpretation. For the additional secondary outcomes of uptake of family planning and exclusive breastfeeding at 6 months, a logistic mixed-effects model will be used. Infant outcomes in child development and nutrition will use linear mixed-effects models with arm, time and arm by time interaction in the model.

Power was estimated for the two primary outcomes, infant receipt of all recommended vaccines and mother’s depression. We plan to have 96 postpartum/well-child groups (48 per arm) with 10 dyads in each group. We assumed a 20% attrition rate and an ICC of 0.02 for infant/mothers within groups derived from our pilot work. This ICC reflects the primary source of clustering in our design. For infant vaccine, we used data from the 2015–2016 Malawi Demographic and Health Survey to estimate the rate in the control group at 72% completing all recommended vaccines [30]. With alpha of 0.05 and total *N* = 768, we have power of 0.87 to detect a significant difference if the vaccine rate in the intervention group is 10% higher (82%). For mother’s depression, we used results from a systematic review of the prevalence of perinatal depression in Malawi to estimate depression in the control group at 19.2% [31]. With an alpha of 0.05 and total *N* = 768, we have a power of 0.96 to detect a significant difference if the depression rate in the intervention group is 10% lower (9.2%). In sensitivity analyses, we also examined power when the ICC is estimated at the clinic level (ICC = 0.015 based on prior data). Power is 0.92 for mother’s depression and 0.82 for vaccine rate.

#### 2.6.2. Aim 2: Explore the Health and Health-Related Social Needs That Arise in the 12 Months After Birth for Both Women and Their Infants Who Experience Complex Postnatal Health or Social Issues

Recruitment. Sixteen women from the control group and 16 women from the intervention group (*n* = 32) who experienced a complication during childbirth (e.g., postpartum hemorrhage) and scored high on the EPDS at 6 or 12 months or on another complex health issue postpartum will be asked to participate in a 60 min interview to document their care pathway experience. This purposive sampling strategy focuses on women and infants with complex health and/or social needs, as they are most likely to interact with the health system in ways that reveal gaps, facilitators, and barriers to care-seeking in the 12-month postpartum period. If interested, they will be screened for eligibility. To respect family and work responsibilities, the members of the research team will schedule the interview at convenient times. While we anticipate that 32 interviews will allow us to reach saturation (defined here as the point when no new themes emerge), interviewing will continue until saturation is reached [32].

Data collection procedures. A trained member of the study team will conduct all interviews. After consent is reaffirmed, each interview will be audio-recorded and conducted in either English or the local language, Chichewa, based on the participant’s preference, and translated into English if conducted in Chichewa. A trained research assistant from the study team will be responsible for transcribing and translating the audio recordings. A certificate of translation will be provided to ensure the integrity of the translated data. The transcribed interviews will be stored, managed, and analyzed using Dedoose software (version 10.1.13), a qualitative data management program. Study notes will be used to track interview observations conducted immediately after each interview. Interviews will be identified by date, place, and data type, transcribed verbatim, reviewed for accuracy, de-identified by substituting pseudonyms for names of individuals, and stored as computer files. The interviewer will listen to the original recordings to verify the accuracy of the typed transcripts. Individual interview guides and interviews will be translated using a standard team approach to translation [33]. Transcripts will be imported into Dedoose and we will follow recommended strategies to code, store, and retrieve data [32].

Analyses for Aim 2. Notes and transcripts will be collated to provide summaries of postpartum and infant health and health-related social needs identified in the 12 months after birth by the participant and the care that was received or was desired during that time. A conventional content analysis approach will be used to analyze interviews, and a category system will be created [34]. The team will create a category system based on the health and related social needs in the first year postpartum, coding themes using the interviews as the unit of analysis [35]. New codes will be added as needed. The researchers will assess the rigor of the data through the four dimensions criteria: credibility, dependability, confirmability, and transferability [32]. The data gained through the analysis will be used to provide a greater understanding of the care pathways that happen in the first year postpartum and the identified needs of women and infant dyads. The data will also provide a greater understanding of the differences in care experiences between usual (individual) and group care.

#### 2.6.3. Aim 3: Identify Clinic- and Patient-Level Implementation Facilitators and Barriers

We will identify clinic- and patient-level implementation facilitators and barriers to facilitate rapid uptake and ownership should the results support doing so. Therefore, we draw on established implementation frameworks, including Exploration, Preparation, Implementation, Sustainment (EPIS) [36] and the domains and constructs of Proctor et al.’s Outcomes for Implementation Research [37], because these frameworks will allow us to identify features that affect implementation as health systems adopt new evidence-based interventions. EPIS has four implementation phases and delineates the influences of outer (policies, funding, cross-agency cooperation) and inner (organizational capacity, climate, leadership) contexts on implementation processes as well as bridging factors such as the nature of the intervention and the role of innovation [36]. Further, it is established that implementation proceeds more quickly when technical assistance is provided across the steps leading to health system ownership of the intervention. We will use the same Three-Step Implementation Model and Toolkit that allowed us to successfully implement and sustain group antenatal care at seven clinics in Blantyre District, Malawi, since adopting this service delivery model [21]. Step 1, Prepare, includes training facilitators to offer GPWCC with fidelity and providing all clinics with group care kits. Step 2, Rollout, includes technical assistance on site at each clinic to provide tailored support throughout implementation. Step 3, Sustain, involves making decisions about whether to continue offering GPWCC beyond the period of the trial. Together with the bridging (academic–government–health system partnership) and innovation factors (GPWCC fit within each clinic), we draw on climate and organizational readiness theories and measures to explore how these aspects of the clinic relate to interconnections, interactions, linkages, and relationships. Through our partnership, we will address barriers and facilitators to implementation (Exploration) and planned delivery (e.g., trained staff (Preparation) and implement the model with high fidelity, assessing women’s satisfaction, attendance, and services provided (Implementation)) [18]. This approach has been successfully used for implementation of other behavior change and service delivery interventions in clinical and community settings [38,39,40,41]. This will also provide crucial information about the sustainability and reproducibility of GPWCC in other settings.

Recruitment: We will observe and systematically take notes at meetings. Key stakeholders at the Ministry of Health, Zomba District Health Office, and clinic administration (*n* = 16) are interviewed annually. Each clinic’s focal person and group care mentors will be asked to participate in interviews (*n* = 16). A sample of co-facilitators (midwives [*n* = 8] and HSAs [*n* = 8]) will also be asked to participate in interviews to discuss the implementation of group care at their clinics. We hypothesize that we will reach saturation with 16 co-facilitators but will continue to interview until we reach saturation [32]. Eligibility criteria are based on the person’s position at the time of data collection (e.g., the Maternal Health Officer at the Reproductive Health Unit, midwife at participating clinic). Consent is obtained before the first time that data are collected. Persons no longer in the designated position are dropped, and persons coming into these positions are consented to participate at the next data collection point. Women (*n* = 20) who participated in groups across the eight clinics will also be interviewed to discuss their group care experience. Again, we hypothesize that saturation will be reached with 20 women in the intervention group; however, we will continue interviewing until saturation is reached.

Data collection procedures: Data sources include study notes, qualitative data from interviews, structured observations, administrative data (e.g., attendance and visit tracking forms (e.g., services received)), and satisfaction and peer connectedness surveys adapted from group antenatal care administered at the 6-and 12-month time points of the study [21]. A brief form will be completed by the facilitator each time a group visit is held to track the date of the session and the number of participants from the group in attendance, in order to track dose. Data collection begins with the sensitization of stakeholders and continues until our final National Dissemination Conference. All members of the implementation team will take study notes that include observations and discussion details from in-person and phone meetings, and qualitative interviews will capture salient information about implementation successes, challenges, solutions, decision-making, interpersonal interactions, and additional experiences including interpretations and evaluations of evidence. We will assess fidelity to the model with a structured observation fidelity checklist. A sample of two out of six group visits will be observed for each group cohort to monitor fidelity to the model (e.g., whether content is delivered as intended, women are engaged enough to actively participate in group discussions and activities, health care occurs in the group space, etc.). The peer connectedness and satisfaction surveys will provide insights into the experiences of participants related to social support and fidelity to the group care model. See Aim 2 above for procedures for qualitative interviews.

Analyses for Aim 3. Notes and transcripts will be collated to provide summaries of clinic, patient, and provider implementation barriers and facilitators. A conventional content analysis approach will be used to analyze interviews, and a category system will be created [34]. The team will create a category system based on the implementation frameworks mentioned above and code themes using the interviews as the unit of analysis [35]. New codes will be added as needed. The researchers will assess the rigor of the data through the four dimensions criteria: credibility, dependability, confirmability, and transferability [32]. The fidelity observation checklist will be analyzed in total and at the clinic level. For each of the six sessions, the percentage of items meeting the threshold for fidelity will be determined and the mean percentage fidelity across the six sessions computed to obtain a score for each clinic.

#### 2.6.4. Blinding

Due to the nature of the intervention, participants and health care workers cannot be blinded to group assignment. Outcome assessors are aware of clinic allocation; however, primary outcomes are ascertained through standardized instruments (EPDS) and health records (vaccination status), minimizing assessor influence on measurement. Data analysts will conduct primary analyses blinded to allocation where feasible. Protocol deviations will be documented systematically and reported.

### 2.7. Trial Oversight and Monitoring

Trial Registration and Status. This trial was registered on ClinicalTrials.gov (NCT07414901) prior to the enrollment of the first participant. Complete registration details, including registration date, recruitment dates, and current study status, are available at the registry (https://clinicaltrials.gov/study/NCT07414901 (accessed on 11 February 2026)).

Data monitoring: Given the minimal-risk behavioral nature of the intervention, a formal Data Monitoring Committee was not established.

Study discontinuation: This study may be temporarily suspended or prematurely terminated if there is sufficient reasonable cause. Written notification, documenting the reason for study suspension or termination, will be provided by the suspending or terminating party to study participants, investigators, funding agency, and regulatory authorities. Circumstances that may warrant termination or suspension include, but are not limited to: determination of unexpected, significant, or unacceptable risk to participants; demonstration of efficacy that would warrant stopping; insufficient compliance of study staff with the protocol (i.e., significant protocol violations); data that are not sufficiently complete and/or evaluable; determination that the primary endpoint has been met; and determination of futility. The study may resume once concerns about safety, protocol compliance, and data quality are addressed and the funding agency, sponsor, IRB, or other relevant regulatory or oversight bodies are satisfied.

Adverse Event Reporting. Serious adverse events are defined and reported according to Johns Hopkins Medicine IRB requirements.

Protocol Amendments. Any important protocol modifications will be submitted for approval to both the Johns Hopkins Medicine IRB and the Kamuzu University of Health Sciences Research Ethics Committee, and the trial registry will be updated accordingly.

## 3. Discussion

Group care that integrates postpartum and well-child care is a paradigm-shifting health care service delivery model that has the potential to improve maternal and infant health outcomes in low-resource settings. The group care service delivery model is a transformative approach to integrating postpartum and well-child care in a respectful manner. Innovations focused on the health system level can provide crucial opportunities to mitigate maternal and infant health determinants and, for those with childbirth and/or postpartum complications, identify and treat conditions that could ultimately lead to maternal or infant death or impair quality of life [42]. By providing health care in a group space and longer visits that include interactive learning and community building, this model can address whole person health, including the social drivers of health, to improve maternal health and well-being, which we hypothesize will accelerate sustainable reductions in maternal and infant morbidities. Preliminary evidence suggests that this model has promise for improving multiple maternal and child health outcomes that accumulate and diffuse. The model increases the time for assessment of both health and health-related social needs and provides quality preventive health care while developing a social support system for women and families [43,44,45]. This improved quality of care has the potential to reduce maternal and infant morbidities and mortality without a significant increase in costs/resources and potential long-term cost savings [46].

This study is innovative in proposing one of the first rigorous studies of a theory-based GPWCC model co-designed in partnership with health care providers and postpartum women in a low-resource health system. To the best of our knowledge, this is the first study to conduct a clinical trial on group care in the postpartum period in an LMIC. The findings will add to the growing body of evidence of GPWCC. The findings from this study can be further adapted and tested throughout Malawi and disseminated and adapted to health care systems in diverse low-resource contexts. During training and rollout of the GPWCC model, our Three-Step Implementation Model simplifies EPIS and other implementation models into an accessible tool that health systems and clinics can use [38,39,40,41]. The Three-Step Implementation Model and the associated toolkit incorporate perspectives from diverse stakeholders to position each clinic to meet goals while increasing the likelihood of uptake and sustainability of the group care model. Each of the eight clinics assigned to the group care arm will follow all three implementation steps (prepare, rollout, and sustain), with extensive initial support from research team members during preparation and rollout. Support decreases gradually as clinics gain the experience to sustain group care. If successful, the clinics will have the option of offering group care as an alternative model of care. This pragmatic implementation approach and associated evidence-based strategies support implementation and future ownership by building capacities through step-by-step procedures as we prepare, roll out and support future sustainment of GPWCC should data show this model of care to be effective for improving maternal and infant health.

This trial evaluates the effectiveness of the integrated GPWCC package as a whole, rather than isolating the independent effect of receiving care in a group format. Findings should be interpreted as the effect of the complete intervention as implemented, recognizing that differences between arms include both the model of care delivery and the structure and frequency of encounters.

This study has limitations. The number of randomized clusters (16 clinics) may limit statistical power for detecting smaller effect sizes, and findings may not generalize beyond Zomba District or health systems with similar infrastructure and staffing. Despite geographic separation of clinics, contamination remains possible if participants seek care elsewhere or share information across sites. Blinding of participants and providers is not feasible given the nature of the intervention, and outcome assessors are aware of clinic allocation; however, reliance on health records and standardized instruments for primary outcomes reduces potential bias. Some secondary outcomes rely on self-report, which may be subject to recall or social desirability bias. The intervention arm receives more frequent and longer visits than usual care, making it difficult to isolate specific mechanisms of effect. Finally, loss to follow-up over the 12-month study period may introduce attrition bias, though we have powered the study assuming 20% attrition.

## 4. Conclusions

Group care models can be optimized to provide preventive health care to the woman/infant dyad in the critical year postpartum and address health and social needs by providing high-quality health care. Through providing health care in a group space, offering interactive learning and community building, group care has the potential to increase recommended childhood vaccinations to prevent disease, improve the nutritional status of infants and their development, reduce postpartum depression, anemia and hypertension, and increase rates of family planning uptake, which all can work together to improve family and community health. This rigorous study will have important public health policy implications by creating an evidence-based foundation for other low-resource settings to build on. Results will enhance our understanding of postpartum health beyond 6 weeks and provide clinical evidence for improved maternal health care in the first year postpartum.

## Figures and Tables

**Table 1:** Session (time postpartum) and associated health promotion topics for facilitated interactive learning.

Session		Health Promotion Topics
1	6 wks	Joys & challenges of a new baby Discomforts and danger signs Physical & emotional adjustments after birth Breastfeeding Sexual activity/family planning
2	10 wks	Immunizations Nutrition for the family Sexual health (e.g., STIs, HIV, PMTCT) Intimate partner violence/relationship issues
3	14 wks	Growth monitoring & developmental milestones Infant feeding: preparing to introduce solid foods Mental health/postpartum depression
4	6 mo	Infant feeding: solids and textures Growth & development Disease prevention
5	9 mo	Management of common childhood illnesses Partner involvement Maternal physical health: a focus on hypertension
6	12 mo	Growth & development: Milestones Nutrition for the family Maternal health maintenance (e.g., breast exam, cervical & ovarian cancer)

## Data Availability

This protocol paper does not report any data. Data sharing is not applicable to this article.

## Abbreviations

The following abbreviations are used in this manuscript:

EPDS	Edinburgh Postnatal Depression Scale
EPIS	Exploration, Preparation, Implementation, Sustainment
GPWCC	Group Postpartum and Well-Child Care
Hb	Hemoglobin
HSA	Health Surveillance Assistant
ICC	Intraclass Correlation Coefficient
IRB	Institutional Review Board
LMIC	Low- and Middle-Income Country
MDAT	Malawi Developmental Assessment Tool
WHO	World Health Organization
